# Reviewing the Safety of Providing Out-of-Operating-Room Anesthesia for Acute Burns in Pediatric Patients

**DOI:** 10.1093/jbcr/irag118

**Published:** 2026-07-08

**Authors:** Sabrina Yang, Sara Faour, Farrukh Munshey, Tobias Everett, Charis Kelly, Eduardo Gus, Joel Fish

**Affiliations:** Faculty of Medicine, University of Ottawa, Ottawa Ontario K1H 8M5, Canada; Division of Plastic and Reconstructive Surgery, The Hospital for Sick Children, Toronto, Ontario M5G 1X8, Canada; Department of Anesthesia and Pain Medicine, The Hospital for Sick Children, Toronto, Ontario M5G 1XB, Canada; Department of Anesthesia and Pain Medicine, The Hospital for Sick Children, Toronto, Ontario M5G 1XB, Canada; Division of Plastic and Reconstructive Surgery, The Hospital for Sick Children, Toronto, Ontario M5G 1X8, Canada; Division of Plastic and Reconstructive Surgery, The Hospital for Sick Children, Toronto, Ontario M5G 1X8, Canada; Department of Surgery, Temerty Faculty of Medicine, University of Toronto, Toronto, Ontario M5T 1P5, Canada; Division of Plastic and Reconstructive Surgery, The Hospital for Sick Children, Toronto, Ontario M5G 1X8, Canada; Department of Surgery, Temerty Faculty of Medicine, University of Toronto, Toronto, Ontario M5T 1P5, Canada

**Keywords:** pediatric burns, burn wound care, procedural sedation, anesthesia

## Abstract

Pain management is a cornerstone of acute pediatric burn care, and burn wound care frequently requires sedation or anesthesia to optimize patient comfort and procedural success. Anesthesia delivered outside of the operating room (OR) has been shown to be a safe and effective alternative to OR-based care; however, eligibility has traditionally been restricted to older and larger children. Following a quality care audit in 2022, eligibility for anesthesiologist-administered out-of-OR anesthesia at our tertiary pediatric burn center was expanded to include children aged 8-12 months and/or weighing 8-10 kg. The objective of this study was to compare key quality and safety indicators between patients meeting expanded eligibility criteria and those meeting historical criteria (>10 kg and/or 12-24 months) undergoing burn dressing changes in a general anesthesia-ready satellite anesthesia room. This single-center retrospective study included patients treated between January 2022 and January 2024. Outcomes included procedure duration, anesthetic technique, airway management, anesthesia-related complications, and postprocedure disposition. A total of 211 patients undergoing 296 procedures were included. Burn characteristics were similar between groups, with scald injuries and partial-thickness burns predominating. No episodes of oxygen desaturation, hemodynamic instability, airway obstruction, or difficult mask ventilation were observed. One episode of laryngospasm occurred in the expanded group and resolved with conservative airway management. Procedure duration was shorter, while time to discharge was slightly longer, in the expanded group. These findings suggest that expanding eligibility criteria for out-of-OR anesthesia in pediatric patients with burn injuries is safe and feasible, without compromising procedural efficiency or outcomes.

## INTRODUCTION

Burn injuries are one of the most common types of traumatic injuries in children across North America, with more than 3000 pediatric patients admitted to burn centers each year for acute care.^1^ Pain management is a cornerstone of acute pediatric burn care, and its role in recovery is well described.^2^ In fact, inadequate pain management has been directly linked to posttraumatic stress disorder among pediatric patients with burn injuries of all ages within months of treatment, and pain can delay recovery.^3^^,^^4^ Pain management associated with burn wound care remains one of the most challenging aspects of treatment.^5^ These injuries often require frequent dressing changes, which can be physically distressing and have lasting psychological and emotional consequences. Effective analgesia and, when required, deep sedation or general anesthesia are therefore critical to optimizing patient comfort, procedural success, and long-term well-being.^6^ Beyond improving patient comfort, adequate sedation is also essential for appropriate burn management—ensuring optimal wound cleaning, thorough debridement, and accurate assessment.^7^

While general anesthesia in the operating room (OR) has traditionally been the predominant solution for pediatric burn care, out-of-OR facilities providing anesthesia or sedation have been shown to offer a safe and effective alternative.^5^^,^^8^ Research suggests that outpatient wound care can achieve low complication rates and should be considered the standard of care for healthy children with appropriate burn size and adequate home support.^9^ At our tertiary-care institution, pediatric anesthesiologist-administered general anesthesia (GA) or deep sedation for burn dressing changes outside the OR had historically been limited to patients over 10 kg in weight and older than 12 months. Following a quality care audit in 2022, these criteria were expanded to include children weighing more than 8 kg and/or older than 8 months, with the goals of accommodating out-of-OR care for a higher volume of acute burn cases and reducing costly and unnecessary use of OR resources.

The primary objective of this study is to compare key quality and safety indicators (ie, procedure duration, anesthesia technique, procedural and anesthesia-related complications, postprocedure disposition) between patients meeting the expanded criteria (8-10 kg and/or 8-12 months) and those meeting historical criteria (>10 kg and 12-24 months) who received dressing changes in out-of-OR treatments rooms. This comparison aims to assess the impact of the expanded eligibility on the safety and effectiveness of out-of-OR anesthesia for dressing changes in pediatric patients with burn injuries.

## METHODS

This single-site retrospective study was conducted at an American Burn Association-verified burn unit at a tertiary pediatric hospital in Ontario, Canada and included patients treated between January 1, 2022 and January 1, 2024. Ethics approval was obtained from our institution (study # 1000081794) prior to the commencement of this study with a waiver of consent due to the retrospective design.

All burn procedures are carried out in a GA-ready treatment room (“satellite anesthesia room”) where anesthesia assistants (AAs) and/or pediatric anesthesiologists provide varying levels of sedation to all patients. The satellite anesthesia room is located remotely from the main OR.

Eligible participants included children aged 8-12 months and/or weighing 8-10 kg (expanded criteria group) as well as those aged 12-24 months and/or weighing greater than 10 kg (historical criteria group) who underwent burn wound treatment (ie, dressing change) with deep sedation or GA in the satellite anesthesia room with the presence of a pediatric anesthesiologist. Cases managed exclusively with AA-administered procedural sedation were excluded because AAs provide sedation within predefined protocols and dosing limits for selected lower-acuity cases. Accordingly, only procedures performed under the supervision of a pediatric anesthesiologist were included in the analysis.

Sedation or GA may be used at multiple stages of acute burn care depending on injury severity and clinical needs. In our institution, out-of-OR anesthesia is commonly used for definitive dressing application, dressing takedown and reassessment, postoperative graft management, and other burn wound care procedures requiring patient immobility, comfort, or extensive wound manipulation.

Burns were initially dressed with a non-adherent dressing until definitive burn depth assessment could be performed. At subsequent dressing changes, wounds were cleansed and managed according to burn depth, with superficial partial-thickness burns typically treated with Mepilex Ag and deeper burns treated with silver-based antimicrobial dressings such as Acticoat or Exsalt, in accordance with institutional protocols.

Data were extracted from medical records in EPIC (Epic Systems Corporation, Verona, WI, USA*)* and included patient demographics, burn injury characteristics, medication and sedation details, and adverse events or complications related to anesthesia or sedation. All data were managed securely using a REDCap (Research Electronic Data Capture; Vanderbilt University, Nashville, TN, USA) electronic data capture tool hosted by our institution.

Descriptive and inferential statistics were used to summarize demographic characteristics, burn treatment procedures, and sedation safety events. Event rates and quality indicators were compared between the expanded criteria group (8-10 kg/8-12 months) and the historical criteria group (>10 kg/12–24 months). Some patients underwent more than one burn dressing change under sedation during the study period. Accordingly, analyses were conducted at both the patient level (for demographic and injury characteristics) and the procedure level (for medication use, airway management, and recovery parameters). All eligible cases during the study period were included as a convenience sample. Continuous variables were non-normally distributed and summarized as medians with interquartile ranges; comparisons were made using the Wilcoxon rank-sum test. Categorical variables were summarized as frequencies and proportions and compared using the chi-square test or Fisher’s exact test when expected frequencies were fewer than 5. All tests were 2-sided, with statistical significance defined as *P* < .05.

## RESULTS

A total of 33 patients with 45 procedures met the expanded criteria group, and 178 patients with 251 procedures met the historical criteria group. As expected, patients in the expanded group were younger and weighed less than those in the historical group, with a median of 10.5 months vs 18.0 months and 9.9 kg vs 11.5 kg, respectively, while sex distribution was similar between groups (Table 1).

**Table 1:** Demographic and Injury Characteristics of Pediatric Burn Patients by Study Group

**Patient demographics**	**Expanded group** **(*n* = 33)**	**Historical group** **(*n* = 178)**	***P*-value**
**Age in months, median (IQR)**	10.5 (9.0-12.0)	18.0 (15.0-21.0)	<.0001^*^
**Weight in kg, median (IQR)**	9.9 (9.3-10.8)	11.5 (10.5-12.6)	<.0001^*^
**Sex, *n* (%)**			
Female	12 (36.36)	84 (47.19)	
Male	21 (63.64)	94 (52.81)	
**TBSA %, median (IQR)**	3.2 (1.0-5.0)	3.0 (1.3-5.3)	.8475^*^
**Burn etiology, *n* (%)**			
Scald	25 (75.76)	143 (80.34)	.5486^†^
Contact	8 (24.24)	35 (19.66)	.4832^†^
Flame	0 (0)	1 (0.56)	>.9999^‡^
**Area(s) burned, *n* (%)**			
Head	6 (18.18)	60 (33.71)	.0772^†^
Neck	2 (6.06)	29 (16.29)	.1806^‡^
Chest	3 (9.09)	89 (50.0)	**<.0001^‡^**
Abdomen	11 (33.33)	13 (7.30)	**<.0001^†^**
Back	2 (6.06)	13 (7.30)	>.9999^‡^
Groin	1 (3.03)	0 (0)	.1564^‡^
Buttock	1 (3.03)	2 (1.12)	.4012^‡^
Leg	8 (24.24)	22 (12.36)	.1275^†^
Foot	2 (6.06)	11 (6.18)	>.9999^‡^
Arm	18 (54.55)	97 (54.49)	>.9999^†^
Hand	16 (48.48)	54 (30.34)	.0669^†^
**Burn depth, *n* (%)**			
Superficial	0 (0)	1 (0.56)	>.9999^‡^
Partial thickness	31 (93.94)	173 (97.19)	.6681^†^
Full thickness	4 (12.12)	26 (14.61)	>.9999^‡^
Not documented	0 (0)	2 (1.12)	>.9999^‡^

Abbreviation: IQR, interquartile range.

“Area(s) burned” represent the proportion of patients with burns at each anatomical location, and burn depth reflects the deepest documented burn for each patient; because patients may have multiple burn sites with varying depths, percentages may not sum to 100%. Values in bold represent items that reached statistical significance. Values in bold represent items that reached statistical significance.

^*^Wilcoxon rank-sum test.

^†^Chi-square test.

^‡^Fisher’s exact tests.

Scald burns were the most common etiology in both cohorts (expanded: 76%, historical: 80%), followed by contact burns (expanded: 24%, historical: 19%). One flame burn was recorded in the historical group (0.56%). No electrical or chemical burns were recorded in this cohort. The median TBSA was similar with values of 3.2% and 3% in the expanded and historical groups, respectively. Partial-thickness burns predominated (expanded: 94%, historical: 97%), followed by full-thickness burns (expanded: 12%, historical: 15%) and upper limbs (arm and hand) were the most frequently affected site in both cohorts (Table 1).

The mean procedure duration was slightly shorter in the expanded group (58.82 ± 16.92 min vs 63.13 ± 24.78 min; *P* = .0033) (Table 2). Premedication was administered significantly less frequently in the expanded group (27% vs 65%; *P* < .0001) compared to the historical group. Midazolam was the only agent used, primarily via the intranasal route, with no administration of ketamine, dexmedetomidine, or other sedatives (Table 2). Intraprocedural sedation primarily involved intravenous route in the expanded group (100%) and intravenous and intranasal in the historical group (97% and 4%, respectively). Morphine and propofol were administered significantly more frequently in the expanded group (58% vs 32%, *P* = .0019; 73% vs 54%, *P* = .0260), whereas fentanyl was administered significantly less frequently (24% vs 48%, *P* = .0053) when compared to the historical group (Table 2). Airway management predominantly consisted of anesthesia face mask oxygen or nasal prongs, with endotracheal intubation being rare. Endotracheal tube intubation was documented in 3 cases (expanded: 1 [2%], historical: 2 [0.8%]), all of which were reported as elective (Table 2).

**Table 2:** Medication and Airway Management in Pediatric Burn Patients by Study Group

**Sedation variables**	**Expanded group** **(*n* = 45)** *n* (%)	**Historical group** **(*n* = 251)** *n* (%)	***P*-value**
**Premedication route**			
Oral	0 (0)	10 (3.98)	.3691^‡^
Intranasal	12 (26.67)	144 (57.37)	**.0003^†^**
Intravenous	0 (0)	6 (2.39)	.5957^‡^
Not documented	0 (0)	2 (0.80)	>.9999^‡^
**Premedication given**			
Midazolam	12 (26.67)	162 (64.54)	**<.0001^†^**
**Intraprocedural med route**			
Intranasal	0 (0)	11 (4.38)	.3823^‡^
Intravenous	45 (100)	243 (96.81)	.2247^†^
**Intraprocedural med given**			
Fentanyl	11 (24.44)	121 (48.21)	**.0053^†^**
Hydromorphone	0 (0)	5 (1.99)	>.9999^‡^
Morphine	26 (57.78)	81 (32.27)	**.0019^†^**
Ketamine	32 (71.11)	171 (68.13)	.8238^†^
Midazolam	6 (13.33)	39 (15.54)	.8777^†^
Dexmedetomidine	1 (2.22)	16 (6.37)	.4850^‡^
Propofol	33 (73.33)	136 (54.18)	**.0260^†^**
Lidocaine	0 (0)	1 (0.40)	.4850^‡^
Not documented	0 (0)	1 (0.40)	.4850^‡^
**Airway management**			
Endotracheal intubation	1 (2.22)	2 (0.80)	.3914^‡^
Laryngeal mask airway	0 (0)	7 (2.79)	.5999^‡^
Oropharyngeal airway	1 (2.22)	6 (2.39)	>.9999^‡^
Nasal prongs	10 (22.22)	48 (19.12)	.7808^†^
Anesthesia face mask oxygen	40 (88.89)	212 (84.46)	.5884^†^
None	0 (0)	15 (6.0)	.1387^‡^
**Vascular access considerations, *n* (%)**			
Difficult IV access	3 (6.67)	22 (8.76)	.7784^‡^
**Intraprocedural med complications, *n* (%)**			
Laryngospasm	0 (0)	1 (0.4)	>.9999^‡^
None	42 (93.33)	229 (91.24)	.8610^†^
**Procedure duration (min) mean ± SD**	58.82 ± 16.92	63.13 ± 24.78	.0033^*^
**Disposition**			
Discharge home	45 (100)	249 (99.2)	>.9999^†^
Continued admission	0 (0)	2 (0.8)	>.9999^‡^
**Time to discharge postprocedure (min) mean ± SD**	39.22 ± 14.04	34.3 ± 19.55	.0141^*^

Abbreviation: IQR, interquartile range.

“Premedication,” “intraprocedural medication,” and “airway management” categories represent the proportion of patients who received each intervention. Because individual patients may have received multiple medications or airway modalities, percentages may not sum to 100%. Values in bold represent items that reached statistical significance.

^*^Wilcoxon rank-sum test.

^†^Chi-square test.

^‡^Fisher’s exact tests.

Challenges with intravenous (IV) access were noted in 3 cases (6.7%) and 22 cases (8.8%) in the expanded and historical group, respectively. These were anticipated difficulties in obtaining IV access rather than procedural complications. Sedation was well tolerated across both groups, with no episodes of oxygen desaturation, bradycardia, hypotension, airway obstruction, or difficult mask ventilation requiring intervention (Table 2). One patient in the expanded group experienced laryngospasm, which resolved with airway maneuvers and positive-pressure mask ventilation after deepening sedation with a propofol bolus. The patient was also given salbutamol by mask, and the event did not recur during subsequent dressing changes.

Postprocedure, 2 patients had unexpected admissions, both of which occurred in the historical group and were related to the underlying burn injury rather than to sedation or anesthesia. One patient with extensive scald burns developed a burn wound infection and staph scalded skin syndrome requiring inpatient management. The second patient, transferred from a community hospital, was admitted for 1 week for observation and treated with intravenous cefazolin after developing a fever. Time to discharge among outpatients was longer in the expanded group (39.22 ± 14.0 min vs 34.33 ± 19.6 min; *P* = .0141) when compared to historical group (Table 2).

## DISCUSSION

This study demonstrates that expanding eligibility criteria for satellite anesthesia sedation in pediatric patients with burn injuries to include children aged 8-12 months and/or weighing 8-10 kg is both safe and feasible. This expansion did not compromise procedural efficiency or patient outcomes. By broadening eligibility, our program was able to provide timely burn wound care for a more vulnerable patient group while reducing reliance on limited OR resources.

Consistent with previous reports supporting the safety of outpatient sedation in pediatric burn care, our analysis found no significant increase in intraprocedural complications, airway interventions, or adverse events following expansion.^5^^,^^8^ Challenges with intravenous access occurred at comparable rates between groups. In the historical cohort, two patients required unplanned admission—one for burn wound infection and subsequent scalded skin syndrome, and another for observation and IV antibiotics after developing a postprocedure fever. All other patients were safely discharged home, and both admissions were unrelated to the sedation or anesthetic procedure.

Procedure duration was modestly shorter in the expanded cohort, although the absolute difference between groups was small and unlikely to be clinically meaningful despite reaching statistical significance. A likely contributor is that younger children often have proportionally smaller burned areas, making each component of the dressing-change process—such as cleansing, assessing the wound bed, and applying topical agents or dressings—less time-consuming than in older children with larger or more complex injuries. Taken together, these considerations may explain the modest difference observed, though its practical impact remains minimal.

Interestingly, despite their younger age, patients in the expanded group were administered premedication less frequently. Developmental differences in distress and separation behaviors may explain this finding. Infants under 1 year of age are less prone to separation anxiety and are often easily soothed by caregivers, reducing the need for pharmacologic anxiolysis.^10^ In contrast, toddlers and preschool-aged children, who make up most of the historical cohort, are more susceptible to separation anxiety and distress during induction, often necessitating sedative premedication.^10^ Consistent with this, our institution has a longstanding practice of avoiding premedication in infants under one year of age.

Conversely, time to discharge was slightly longer in the expanded group. Although intravenous sedation onset may not differ meaningfully between age groups, younger infants often experience more prolonged recovery due to age-related pharmacokinetics, necessitating extended monitoring in the recovery phase. This is consistent with prior evidence indicating that younger children may experience comparatively longer recovery periods following intravenous sedation or anesthesia, even when induction is rapid.^11^ Pharmacologic factors may also have contributed to these patterns. Patients in the expanded group received morphine more frequently than those in the historical cohort, and morphine exhibits slower clearance in neonates and young infants due to immature hepatic glucuronidation and renal excretion pathways.^12^ Propofol, while fast acting, may redistribute more slowly in smaller infants, prolonging residual effects.^13^ Differences in weight-based dosing (mg/kg)—data not collected in this study—may have also influenced recovery duration. In addition to these physiologic considerations, younger infants are often observed more cautiously before discharge, which may further account for the modest difference seen between groups. Taken together, these developmental, pharmacologic, and practice-related factors likely contributed to the slightly longer discharge times in the expanded cohort; however, the absolute difference was small and unlikely to carry meaningful clinical implications.

The epidemiologic profile of burns in this study aligns with established literature, with scalds representing the most common etiology and partial-thickness burns predominating.^14^ Upper limb involvement was also frequent, reflecting typical patterns seen in young children. Beyond these distributions, the generalizability of our findings is supported by the fact that they are derived from routinely collected clinical data in a high-volume pediatric center, providing a realistic representation of real-world practice.

Our results have practical implications for high-volume burn centers, where OR resources are often constrained. Expanding satellite anesthesia eligibility criteria can reduce delays in wound care, facilitate timely dressing changes, and improve pain and anxiety management without compromising safety. These findings may also be helpful for settings that lack a dedicated sedation suite. In such environments, procedural sedation for minor burn wound care is often administered using agents like ketamine or fentanyl in emergency departments, and our data may help guide decisions about which patients could safely undergo more structured out-of-OR sedation when the necessary expertise and monitoring are available. Broader adoption of such protocols may enhance patient-centered outcomes while optimizing institutional efficiency.

Several limitations should be acknowledged. The retrospective design introduces the possibility of selection bias and limits the assessment of patient-reported outcomes, including pain, psychological distress, and satisfaction. The relatively small size of the expanded cohort (*n* = 45) also reduces the sensitivity to detect rare complications. Moreover, we did not evaluate long-term outcomes such as wound healing or scarring, which are important considerations in this population. Prospective, multicenter studies with larger sample sizes are needed to validate our findings. Future investigations examining the economic impact of expanded out-of-OR anesthesia eligibility, including OR utilization and associated healthcare costs, would also be valuable.

Finally, it is important to acknowledge that as a tertiary care institution with extensive experience in pediatric anesthesia, our established infrastructure and familiarity with managing infants likely facilitated the safe implementation of these expanded criteria. In settings with less experience and familiarity with pediatric anesthesia, the comfort of performing procedures outside of the OR might vary. Nonetheless, our findings support the notion that although individual hospital criteria vary, acute burn care procedures (ie, dressing changes, graft takedown) can be safely performed in younger and smaller children in locations outside the OR.

### Conclusion

In summary, this study provides evidence that expanding eligibility criteria for satellite anesthesia sedation to include younger and lower-weight pediatric patients with burn injuries is safe, effective, and associated with outcomes comparable to historical standards. Developmental physiology likely contributed to the observed small differences in procedure duration, premedication requirements, and discharge times. These findings support the broader integration of out-of-OR anesthesia protocols in pediatric burn care and highlight opportunities to optimize both resource utilization and patient-centered care.

## Notes

Author contributions: Sabrina Yang (Data curation [lead], Formal analysis [equal], Writing—original draft [lead], Writing—review & editing [equal]), Sara Faour (Conceptualization [equal], Data curation [supporting], Formal analysis [lead], Methodology [equal], Writing—original draft [supporting], Writing—review & editing [equal]), Farrukh Munshey (Writing—review & editing [equal]), Tobias Everett (Writing—review & editing [equal]), Charis Kelly (Writing—review & editing [equal]), Eduardo Gus (Writing—review & editing [equal]), and Joel Fish (Conceptualization [equal], Supervision [lead]).

Funding: None declared.
